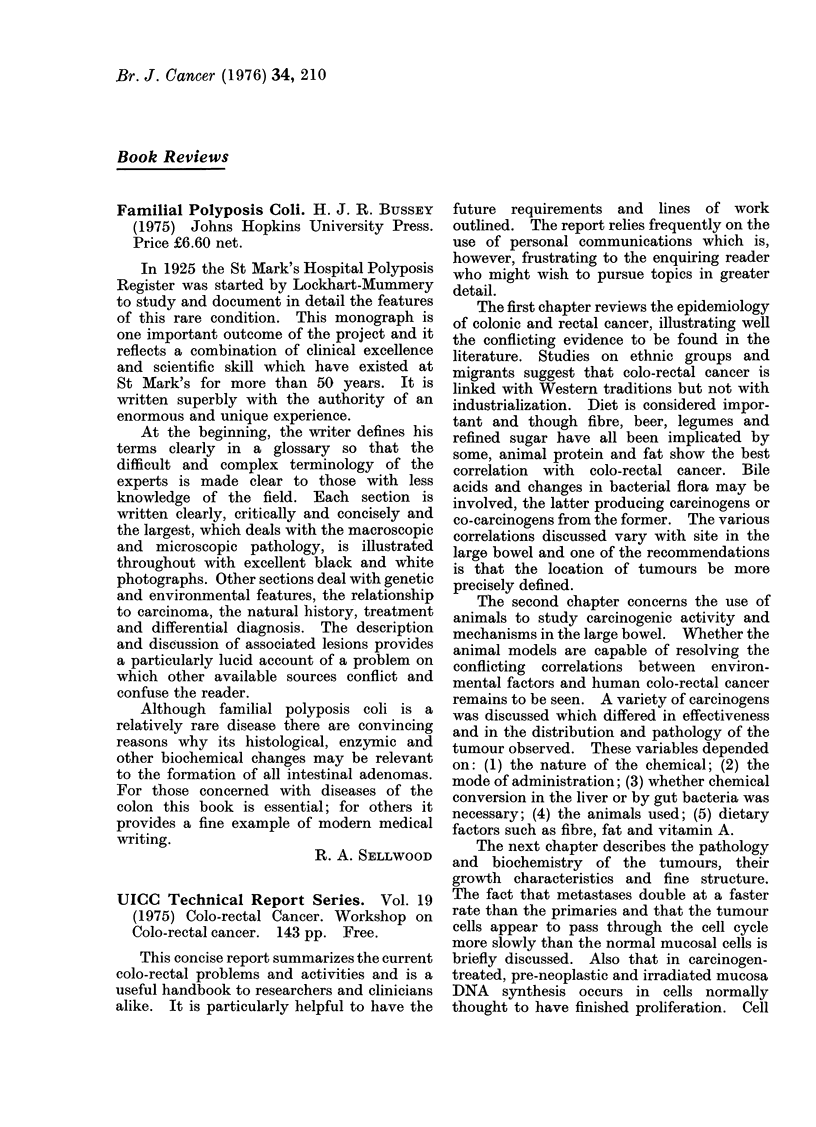# Familial Polyposis Coli

**Published:** 1976-08

**Authors:** R. A. Sellwood


*Br. J. Cancer* (1976) **34**, 210

### *Book Reviews*

**Familial Polyposis Coli.** H. J. R. BUSSEY (1975) Johns Hopkins University Press. Price £6.60 net.

In 1925 the St Mark's Hospital Polyposis Register was started by Lockhart-Mummery to study and document in detail the features of this rare condition. This monograph is one important outcome of the project and it reflects a combination of clinical excellence and scientific skill which have existed at St Mark's for more than 50 years. It is written superbly with the authority of an enormous and unique experience.

At the beginning, the writer defines his terms clearly in a glossary so that the difficult and complex terminology of the experts is made clear to those with less knowledge of the field. Each section is written clearly, critically and concisely and the largest, which deals with the macroscopic and microscopic pathology, is illustrated throughout with excellent black and white photographs. Other sections deal with genetic and environmental features, the relationship to carcinoma, the natural history, treatment and differential diagnosis. The description and discussion of associated lesions provides a particularly lucid account of a problem on which other available sources conflict and confuse the reader.

Although familial polyposis coli is a relatively rare disease there are convincing reasons why its histological, enzymic and other biochemical changes may be relevant to the formation of all intestinal adenomas. For those concerned with diseases of the colon this book is essential; for others it provides a fine example of modern medical writing.

R. A. SELLWOOD